# Prevalence, Risk Factors, and Fetomaternal Outcomes of Gestational Diabetes Mellitus in Kuwait: A Cross-Sectional Study

**DOI:** 10.1155/2019/9136250

**Published:** 2019-03-03

**Authors:** Zainab Groof, Ghadeer Garashi, Hamid Husain, Shaikhah Owayed, Shaima AlBader, Hawra'a Mouhsen, Anwar Mohammad, Ali H. Ziyab

**Affiliations:** ^1^Department of Community Medicine and Behavioral Sciences, Faculty of Medicine, Kuwait University, Safat, Kuwait; ^2^Biochemistry and Molecular Biology Department, Dasman Diabetes Institute, Kuwait City, Kuwait; ^3^Dasman Diabetes Institute, Kuwait City, Kuwait

## Abstract

**Objective:**

Gestational diabetes mellitus (GDM) is a growing global public health problem that can have short- and long-term health consequences for the mother and the child. Despite its criticalness, many countries still do not have the epidemiological data which could guide them in responding to the problem. Due to the lack of knowledge on GDM and the fact that diabetes and obesity are high in Kuwait, this study sought to estimate the prevalence of GDM and determine its risk factors and outcomes.

**Methods:**

This cross-sectional study enrolled 947 mothers living in Kuwait, who had given birth within the previous four years. Participants were recruited from primary health care clinics and public hospitals. GDM status was self-reported by the mother. Associations between exposures and outcomes were evaluated using logistic regression, and adjusted odds ratios (aORs) and 95% confidence intervals (CIs) were estimated.

**Results:**

Of the 868 mothers with no prior history of diabetes mellitus, 109 (12.6%, 95% CI: 10.4, 14.8) reported having been given a GDM diagnosis during their last pregnancy. The prevalence of GDM increased with maternal age and prepregnancy body mass index. GDM was positively associated with caesarean section delivery (aOR = 1.76, 95% CI: 1.17, 2.66) and fetal macrosomia (aOR = 2.36, 95% CI: 1.14, 4.89).

**Conclusion:**

GDM is prevalent in Kuwait and is associated with poor maternal, fetal, and neonatal outcomes. To date, GDM has received little attention, and there is a need for more research to identify and respond to individual and public health implications of GDM in Kuwait.

## 1. Introduction

Gestational diabetes mellitus (GDM) is a transitory form of diabetes (glucose intolerance) with onset or first recognition during pregnancy. It is a major and growing public health problem in most parts of the world, with a global prevalence of between 2% and 6% (and as high as 20% in high-risk populations) [1–3]. Although accurate data on the magnitude of GDM are not available due to the lack of a universally accepted and adopted diagnostic criteria and screening approaches [4–6], GDM is estimated to affect around 1 in 10 pregnant women worldwide [7]. The incidence of GDM is thought to have grown in concert with type 2 diabetes mellitus (T2DM) and the obesity pandemic [1, 2]. Despite the growing frequency of GDM, many countries have been slow in developing the type of policies and practices needed to mitigate or manage the occurrence of GDM due to the lack of empirical knowledge.

GDM carries short- as well as long-term risks for both the mother and neonate. Complications and health consequences of GDM to the mother include caesarean delivery, postpartum hemorrhage, T2DM, obesity, hypertension, and repeated gestational diabetes in subsequent pregnancies [7, 8]. A meta-analysis has estimated the recurrence rate of GDM to be at 48% and can be as high as 80% among high-risk women [9]. Women with previous history of GDM are at 7-fold higher risk for developing T2DM later in life in comparison to those with no GDM history [10]. Maternal age, ethnicity, obesity, and family history of diabetes are common risk factors for GDM [3, 11]. During pregnancy, fetal macrosomia (abnormally large fetus; birthweight of greater than 4000–4500 g) is associated with GDM, which in turn increases the risk of perinatal mortality, asphyxia, and shoulder dystocia during vaginal delivery [12, 13]. Infants and young children of GDM pregnancies are also at increased risk of early obesity and T2DM [14].

The prevalence of GDM in any population appears to be positively correlated to the prevalence of T2DM in women [15]. Moreover, maternal prepregnancy obesity is a major predisposing factor for GDM [16]. In Kuwait, the prevalence of obesity among women aged ≥20 years has been estimated to be 58.6% [17] and of T2DM is estimated to affect 23.1% of the general population [18]. As a result of the aforementioned burden of obesity and T2DM, it is suspected that GDM could be a major and challenging public health problem in Kuwait. However, the main challenge in mitigating the disease is the lack of epidemiologic information. Contributing to the issue is the lack of consistent hospital records and locally standardized conventional screening recommendations. Therefore to have an estimate of the prevalence of GDM among women living in Kuwait, a cross-sectional study was designed to provide data on the prevalence of GDM and identify its risk factors and outcomes.

## 2. Methods

### 2.1. Study Setting

Kuwait is a small country in the Middle East and North Africa region of the World Bank. It has an area of around 18,000 square kilometers and a total population of around 4.2 million. The population of Kuwait can be divided into two separate communities, namely, citizens of Kuwait (Kuwaitis) and labor migrants/expatriates (non-Kuwaitis). Kuwaiti nationals constitute about 31% (1.3 million) of the total population, of which about 660,000 (51%) are women. Of the 2.9 million expatriates in Kuwait, around 970,000 (33.5%) are women. Approximately 304,000 Kuwaiti and 616,000 non-Kuwaiti women are of reproductive age (aged 15 to 44 years). The majority of the non-Kuwaiti women of reproductive age are of Asian ethnicity (348,000) or Arab ethnicity (208,000). Geographically, the population of Kuwait is distributed over six governorates [19]. National health care is provided free-of-charge to citizens and at minimal cost for noncitizens, whereas private hospitals charge fees for everyone.

### 2.2. Study Design and Participants

A population-based cross-sectional study was conducted by enrolling women (*n* = 947) visiting health care facilities across Kuwait. Specifically, women coming to any of the 29 randomly selected vaccination centers for routine vaccination of their children were invited to participate in the study. Additionally, women were recruited into the study from maternity wards at three major general public hospitals. We restricted enrollment to women who gave birth within the past 4 years since most child vaccinations are completed by the age of 4 years. Mothers were asked to self-complete a study-specific questionnaire that captured the demographic characteristics of the mother and her last born child and inquired about their clinical history and complications during the last pregnancy. Ethical approval for this study was obtained from the Health Sciences Center Ethics Committee for Students Research Projects at Kuwait University on March 26, 2015. Written informed consent was obtained from all study participants. The study was conducted in accordance with the principles and guidelines of the Declaration of Helsinki for medical research involving human subjects.

### 2.3. Ascertainment of GDM

To determine GDM status, mothers were asked the following question: “During your last pregnancy, were you told by a doctor, nurse, or other health care worker that you had gestational diabetes (diabetes that started during your last pregnancy)?”, which was adapted from the Pregnancy Risk Assessment Monitoring System (PRAMS) questionnaire [20]. Mothers who responded positively to the previous question and reported no prepregnancy history of doctor-diagnosed diabetes mellitus were considered to have had GDM.

### 2.4. Ascertainment of Exposure and Pregnancy Outcome Variables

Mothers self-reported their prepregnancy weight in kilograms (kg) and height in centimeters (cm) that were used to calculate body mass index (BMI) by dividing weight in kg by height in meters squared (kg/m^2^). Prepregnancy BMI was categorized as the following: normal (BMI < 25.0), overweight (25.0 ≤ BMI < 30.0), and obese (BMI ≥ 30.0). The underweight group (BMI < 18.5) was analyzed with the normal group due to a small proportion (2.0%, 16/813) of mothers being underweight. Family history of GDM was assessed by inquiring whether the participant's mother and/or sister(s) have ever been diagnosed with GDM by a doctor. Family history of diabetes mellitus was ascertained by asking if the participant's mother, father, and/or sibling(s) have ever been diagnosed with diabetes by a doctor. Moreover, mothers self-reported any prior history of stillbirths and/or miscarriages.

In regard to pregnancy-related complications, mothers were asked about the mode of birth of their last born child (vaginal delivery or caesarean section). Pregnancy-induced hypertension was ascertained by asking “During your last pregnancy, did you suffer from pregnancy-related high blood pressure?” Also, mothers were asked to self-report the birth weight of their last born child in kg (fetal macrosomia: birth weight ≥ 4.0 kg) and whether the child was born before 37 gestational weeks (preterm baby: born before 37 gestational weeks).

### 2.5. Statistical Analysis

All statistical analyses were performed using SAS 9.4 (SAS Institute, Cary, NC, USA). The statistical significance level was set to *α* = 0.05 for all association analyses. To determine whether the analytical study sample (*n* = 868; includes participants who have information on GDM status) is representative of the total enrolled study sample (*n* = 947), we used chi-square (*χ*^2^) tests to compare the proportions of categorical variables across these two samples. Odds ratios (ORs) and their associated 95% confidence intervals (CIs) were estimated using logistic regression models. Unadjusted (crude) associations between several maternal characteristics and GDM status were explored. To obtain adjusted associations and to find a set of predictor variables that gives a model with good fit, a stepwise selection method was applied while using a *p* value of 0.2 as a criterion for variable entry and stay in the model.

Associations between GDM status (exposure variable) and pregnancy-related, fetal, and neonatal outcomes were assessed (i.e., mode of delivery, preterm baby, pregnancy-induced hypertension, and fetal macrosomia). For each outcome variable, unadjusted and adjusted models were evaluated. In the unadjusted models, associations between GDM status and outcome variables were tested without statistically adjusting for the effects of any confounders. In contrast, in the adjusted models, potential confounders (i.e., maternal nationality, age, education level, prepregnancy BMI, family history of GDM, and family history of DM) were simultaneously entered into the logistic regression models. To select potential confounders, manual backward elimination process was applied. Covariates that changed the effect (i.e., OR) of the main exposure of interest (i.e., GDM status) by more than 10% when excluded from the model were considered as possible confounders and were retained in the final model; otherwise, the covariate was not considered as a potential confounder. The aforementioned approach of selecting confounders, referred to as “change-in-estimate” approach, aims at providing a model that controls most or all confounding with a minimal number of variables while not relying on the significance testing of the covariate coefficient [21]. Such an approach could yield a different set of confounders for each investigated outcome variable. Moreover, to determine whether any of the considered covariates is an effect modifier of the exposure-outcome association, interactions were evaluated on a multiplicative scale by including product terms in the regression models.

## 3. Results

### 3.1. Description of Study Sample

The analytical study sample (*n* = 868) and the total enrolled study sample (*n* = 947) were similar with respect to all characteristics under study (Table 1). Of the total study participants, 62.3% were of Kuwaiti nationality. The mean age of the study participants was 30 years (standard deviation: ±6). In regard to prepregnancy BMI, 45.7%, 33.2%, and 21.1% of mothers were within the normal, overweight, and obese BMI ranges, respectively (Table 1). The prevalence of self-reported GDM was estimated to be 12.6% (109/868; 95% CI: 10.4%, 14.8%) in the analytical study sample.

### 3.2. Maternal Characteristics and GDM Status

Associations between maternal characteristics and GDM status is shown in Table 2. Our study indicates that GDM is more prevalent among non-Kuwaiti women (16.5%) than Kuwaiti women (10.2%; Table 2). After adjusting for potential confounders, non-Kuwaiti women were more likely to report having GDM than Kuwaiti women (adjusted OR = 2.77, 95% CI: 1.67, 4.58). The prevalence of GDM increased linearly as maternal age increased, reaching 18.2% among mothers aged 35 years and above. Also, an increasing pattern in the prevalence of GDM was seen with prepregnancy BMI, where 7.9%, 15.7%, and 17.0% of women in the normal, overweight, and obesity categories reported having GDM, respectively (Table 2). Women with family (maternal/sister) history of GDM were more likely to report having GDM than those with no family history of GDM (adjusted OR = 2.70, 95% CI: 1.65, 4.41). Moreover, reporting history of giving stillbirth and/or miscarriage was positively associated with having GDM (adjusted OR = 1.71, 95% CI: 1.06, 2.77; Table 2).

### 3.3. GDM Status and Pregnancy and Neonatal Outcomes

Associations between GDM status (exposure variable) and pregnancy, fetal, and neonatal complications were explored (Table 3). Caesarean section delivery was more common among women who reported suffering from GDM during their last pregnancy compared to those without GDM (adjusted OR = 1.76, 95% CI: 1.17, 2.66). Pregnancy-induced hypertension was found to be more common among mothers affected by GDM (25.9%) as compared to those who did not report GDM (16.2%; adjusted OR = 1.63, 95% CI: 0.97, 2.73). In addition, 20.7% of mothers affected by GDM compared to 14.0% of those without GDM reported a history of delivering a preterm baby (OR = 1.61, 95% CI: 0.97, 2.69). Moreover, maternal GDM was positively associated with fetal macrosomia (adjusted OR = 2.36, 95% CI: 1.14, 4.89; Table 3). Of note, none of the tested statistical interactions between covariates and GDM status on the different outcomes presented in Table 3 showed statistical significance (i.e., *p* values associated with product terms (interactions) were greater than the statistical significance level of *α* = 0.05; data not shown).

## 4. Discussion

This study is the first to estimate the prevalence of GDM in Kuwait and determine associated risk factors and outcomes. Our study estimated that 12.6% of pregnancies in Kuwait are affected by GDM, based on self-reporting. Maternal nationality, advanced age, prepregnancy overweight/obesity, family history of T2DM/GDM, and prior history of giving stillbirth/miscarriage were associated with increased risk of GDM in our study. With regard to GDM-associated outcomes, mothers affected by GDM compared to those with no GDM history were at increased risk of caesarean section delivery, giving birth to preterm baby, pregnancy-induced hypertension, and having a macrosomic (large) baby. Our findings indicate that GDM is prevalent among women in Kuwait and is associated with poor maternal, fetal, and neonatal outcomes.

The estimated prevalence of GDM in Kuwait (12.6%, 95% CI: 10.4%, 14.8%) is similar to estimates found in neighboring countries such as Bahrain (10.1%) [22], UAE (13.3%) [23], Saudi Arabia (15.4%) [24], and Qatar (16.3%) [25]. Comparative data for GDM in the United States [20], Australia [26], and Sweden [27] range from 4.6-9.2%, 4.8-6.7%, and 0.4-1.5%, respectively. The elevated prevalence of GDM in Kuwait reflects the high prevalence of T2DM (23.1%) and obesity (58.6% among women aged ≥20 years) in Kuwait [17, 28].

Several maternal characteristics have been reported to be associated with increased risk of developing GDM, such as maternal ethnicity, age, prepregnancy overweight/obesity, and family history of T2DM. Our study showed that maternal nationality was associated with GDM (Kuwaiti 10.2% vs. non-Kuwaiti 16.5% women). Although specific information on maternal ethnicity/race was not acquired in our study, the disparity in the prevalence between Kuwaiti and non-Kuwaiti women is suspected to be due to different maternal ethnicity/race; most non-Kuwaiti women living in Kuwait are of Asian ethnicity (56.5%), which is known to be a high-risk ethnicity for GDM [29]. Moreover, different social, behavioral, and environmental aspects could explain some of the observed nationality-based differences. On the other hand, the observed associations between advanced maternal age, prepregnancy overweight/obesity, and family history of diabetes mellitus with increased risk of GDM concur with findings from prior studies [1, 3, 16]. Similarly, a body of existing literature support the noted associations between GDM and increased risk of maternal and fetal/neonatal complications (e.g., caesarean section delivery, pregnancy-induced hypertension, preterm baby, and fetal macrosomia) [7, 12, 14, 27]. A prior case-control study conducted in Kuwait showed that women with gestational diabetes compared to those with no diabetes (controls) had higher risk of stillbirth and delivering macrosomic babies [30]; results that are similar to our observations. Although we have tested for statistical interactions (effect modification) between covariates and GDM status on the different outcomes (see Table 3), none of the evaluated interaction terms showed statistical significance.

The wide coverage of enrollment venues across all governorates in Kuwait (29 randomly selected vaccination centers and three general public hospitals) is a major strength to our cross-sectional study. Enrolling study participants from different parts of Kuwait helped maximize the representativeness of our study sample. Possible limitations in our study include the cross-sectional nature of our study design which reduced our ability to infer temporal relationships between exposures and outcomes. Self-reporting of information may also have been subject to some degree of reporting/recall bias. In the current study, GDM status was ascertained based on a self-reported method, which can either overestimate or underestimate the prevalence of GDM. Such variation can be due to the lack of a universal screening methodology for GDM and missed diagnosis. An international survey assessing GDM screening and management practices indicated that there is no consensus on a national guideline nor a screening criteria in Kuwait [31]. However, through personal communications with the heads of obstetrics and gynecology departments at different public/private hospitals/clinics in Kuwait, majority have reported using the one-step 75 g oral glucose tolerance test (OGTT) as recommended by the American Diabetes Association (ADA) for GDM screening and diagnosis at 24–28 weeks of gestation [32]. Nevertheless, some cases of GDM may not have been detected through the current screening and diagnostic strategies. Given the aforementioned challenges, misclassification of GDM status is inevitable; nevertheless, the results on GDM outcomes observed in the current report are similar to prior reports, and the estimated prevalence is similar to regional estimates, which is an indication that probably most of the mothers are well classified into GDM or not. However, future studies using the objective methods of GDM ascertainment are needed to corroborate our findings. Moreover, since pregnancy-induced hypertension was self-reported by mothers, the possibility of misreporting preeclampsia for hypertension or vice versa cannot be excluded.

Maternal prepregnancy overweight/obesity is a major yet a modifiable risk factor for GDM development [3]. Prior studies have indicated that up to 50% of GDM cases can be attributed to maternal prepregnancy overweight/obesity alone [33, 34]. Given the high prevalence of obesity among women of child-bearing age in Kuwait (58.6%) and its critical role in the development of GDM, there is an urgent need to develop national strategies that aim at mitigating the overweight/obesity epidemic in Kuwait. Also, better understanding of the magnitude and risk factors of GDM in Kuwait is needed to develop preventive strategies that aim at improving maternal and child health and mitigate GDM. This investigation is the first to provide much needed long overdue epidemiological data on the nature of the GDM problem in Kuwait.

## 5. Conclusions

Our study found that 12.6% of women in Kuwait are affected by GDM and that mothers and newborns exposed to GDM are at increased risk of adverse health outcomes. To better understand the magnitude of and respond to GDM in Kuwait, there is a need to develop national guidelines, strategies, and polices through first gaining empirical knowledge on the current situation.

## Figures and Tables

**Table 1:** Characteristics of the total study sample and the analytical sample.

	Total study sample (*n* = 947) % (*n*)	Analytical study sample^1^ (*n* = 868) % (*n*)
Nationality		
Kuwaiti	62.3 (590)	62.3 (541)
Non-Kuwaiti	37.7 (357)	37.3 (327)
Age (years)		
<25	16.6 (157)	17.6 (152)
25 – 29	30.0 (283)	30.3 (262)
30 – 34	29.4 (277)	29.2 (253)
≥35	24.0 (226)	22.9 (198)
Missing, n	4	3
Education level		
≤Middle school	5.7 (54)	5.6 (48)
High School	17.6 (166)	16.3 (141)
≥College or university	76.7 (723)	78.1 (676)
Missing (*n*)	4	3
Prepregnancy BMI (kg/m^2^)		
Normal (<25.0)	44.9 (365)	45.7 (343)
Overweight (25.0 – 29.9)	33.2 (270)	33.2 (249)
Obese (≥30.0)	21.9 (178)	21.1 (159)
Missing (*n*)	134	117
Family history of GDM^2^		
Yes	31.0 (291)	28.9 (250)
No	79.0 (649)	71.1 (614)
Missing (*n*)	7	4
Family history of DM^3^		
Yes	59.7 (563)	58.1 (503)
No	40.3 (379)	41.9 (362)
Missing (*n*)	5	3
Stillbirth and/or miscarriage		
Yes	30.7 (286)	29.8 (255)
No	69.3 (645)	70.2 (601)
Missing (*n*)	16	12

GDM: gestational diabetes mellitus; DM: diabetes mellitus; BMI: body mass index. ^1^Analytical study sample is restricted to participants with information on GDM status. ^2^Refers to history of GDM in mother and/or sister of the participant. ^3^Refers to history of DM in mother, father, and/or sibling of the participant.

**Table 2:** Associations between maternal characteristics and gestational diabetes mellitus status.

	GDM % (*n*/total)	Unadjusted OR (95% CI)	Adjusted^1^ OR (95% CI)
Nationality			
Kuwaiti	10.2 (55/541)	1.00 (reference)	1.00 (reference)
Non-Kuwaiti	16.5 (54/327)	1.75 (1.17, 2.62)	2.77 (1.67, 4.58)
*p* value		0.007	<0.001
Age (years)			
<25	6.6 (10/152)	1.00 (reference)	—
25 – 29	10.3 (27/262)	1.63 (0.77, 3.47)	—
30 – 34	14.2 (36/253)	2.36 (1.13, 4.90)	—
≥35	18.2 (36/198)	3.16 (1.51, 6.59)	—
*p* value		0.008	—
Education level			
≤Middle school	27.1 (13/48)	3.03 (1.53, 5.98)	1.78 (0.76, 4.17)
High School	15.6 (22/141)	1.51 (0.90, 2.52)	1.62 (0.92, 2.86)
≥College or university	10.9 (74/677)	1.00 (reference)	1.00 (reference)
*p* value		0.004	0.147
Prepregnancy BMI (kg/m^2^)			
Normal (<25.0)	7.9 (27/343)	1.00 (reference)	1.00 (reference)
Overweight (25.0 – 29.9)	15.7 (39/249)	2.17 (1.29, 3.66)	2.23 (1.29, 3.87)
Obese (≥30.0)	17.0 (27/159)	2.39 (1.35, 4.24)	2.05 (1.11, 3.79)
*p* value		0.003	0.011
Family history of GDM^2^			
No	9.5 (58/614)	1.00 (reference)	1.00 (reference)
Yes	19.9 (50/251)	2.39 (1.58, 3.60)	2.70 (1.65, 4.41)
*p* value		<0.001	<0.001
Family history of DM^3^			
No	8.8 (32/363)	1.00 (reference)	—
Yes	15.1 (76/503)	1.84 (1.19, 2.85)	—
*p* value		0.006	—
History of stillbirth/miscarriage			
No	10.8 (65/602)	1.00 (reference)	1.00 (reference)
Yes	16.1 (41/255)	1.58 (1.04, 2.41)	1.71 (1.06, 2.77)
*p* value		0.033	0.029

GDM: gestational diabetes mellitus; DM: diabetes mellitus; BMI: body mass index; OR: odds ratio; CI: confidence interval. ^1^Stepwise variable selection method was applied to choose the best-fitting model. Variables were selected based on *p* value cutoff of 0.2 for variable entry and stay in the model. Adjusted ORs are presented for the variables that were selected in the stepwise selection process. The variables age and family history of DM were not selected in this selection process. ^2^Refers to history of GDM in mother and/or sister of the participant. ^3^Refers to history of DM in mother, father, and/or sibling of the participant.

**Table 3:** Associations between gestational diabetes mellitus status and pregnancy-related, fetal, and neonatal complications.

Outcome variables	GDM % (*n*)	No GDM % (*n*)	Unadjusted model		Adjusted model	
			OR (95% CI)	*p* value	OR (95% CI)	*p* value
Mode of delivery						
Vaginal	51.9 (56)	67.1 (504)	1.00 (reference)		1.00 (reference)	
Caesarean section	48.1 (52)	32.9 (247)	1.90 (1.26, 2.85)	0.002	1.76 (1.17, 2.66)^1^	0.007
Pregnancy-induced hypertension						
No	74.1 (80)	83.8 (632)	1.00 (reference)		1.00 (reference)	
Yes	25.9 (28)	16.2 (122)	1.81 (1.13, 2.91)	0.014	1.63 (0.97, 2.73)^2^	0.067
Preterm baby (<37 weeks)						
No	79.3 (84)	86.0 (646)	1.00 (reference)			
Yes	20.7 (22)	14.0 (105)	1.61 (0.97, 2.69)	0.068	N/A	
Fetal macrosomia (≥4000 g)						
No	83.2 (79)	93.3 (601)	1.00 (reference)		1.00 (reference)	
Yes	16.8 (16)	6.7 (43)	2.83 (1.52, 5.26)	0.001	2.36 (1.14, 4.89)^3^	0.021

N/A refers to the situation of failing to find a covariate/confounder that changed the OR associated with GDM status of the full model by more than 10%. ^1^Adjusted for maternal nationality. ^2^Adjusted for maternal prepregnancy BMI. ^3^Adjusted for maternal nationality, prepregnancy BMI, and family history of GDM.

## Data Availability

The data used to support the findings of this study are available from the corresponding author upon request.
